# CircDNAJC11 interacts with TAF15 to promote breast cancer progression via enhancing MAPK6 expression and activating the MAPK signaling pathway

**DOI:** 10.1186/s12967-023-04020-x

**Published:** 2023-03-09

**Authors:** Bin Wang, Hang Chen, Yumei Deng, Hong Chen, Lei Xing, Yuping Guo, Min Wang, Junxia Chen

**Affiliations:** 1 Department of Cell Biology and Genetics, Chongqing Medical University, 1 Yixueyuan Road, Chongqing, 400016 People’s Republic of China; 2 Department of Oncology, Daping Hospital of Army Medical University, 10 Changjiang Branch Road, Chongqing, 400042 People’s Republic of China; 3 Institute of Hepatopancreatobiliary Surgery, Chongqing General Hospital, 118 Xingguang Road, Chongqing, 401147 People’s Republic of China; 4 Department of Endocrine and Breast Surgery, The First Affiliated Hospital of Chongqing Medical University, 1 Yixueyuan Road, Chongqing, 400016 People’s Republic of China

**Keywords:** Breast cancer, CircDNAJC11, TAF15, MAPK6, MAPK signaling pathway

## Abstract

**Background:**

Breast cancer (BC) is a common malignant tumor in women worldwide. Circular RNA (circRNA) has been proven to play a critical role in BC progression. However, the exact biological functions and underlying mechanisms of circRNAs in BC remain largely unknown.

**Methods:**

Here, we first screened for differentially expressed circRNAs in 4 pairs of BC tissues and adjacent non-tumor tissues using a circRNA microarray. Functionally, gain- and loss-of-function experiments in vitro and in vivo showed that circDNAJC11 promoted BC cell proliferation, migration, invasion, and tumor growth. Mechanistically, RNA pull-down, mass spectrum, RNA immunoprecipitation, fluorescence in situ hybridization assays, and rescue experiments were executed.

**Results:**

We found that circDNAJC11 was significantly upregulated in triple-negative breast cancer tissues and cells. Clinical data revealed that the high expression of circDNAJC11 was closely correlated with a poor prognosis of BC patients and could be an independent risk factor for BC prognosis. Functionally, gain- and loss-of-function experiments in vitro and in vivo showed that circDNAJC11 promoted BC cell proliferation, migration, invasion, and tumor growth. Mechanistically, RNA pull-down, mass spectrum, RNA immunoprecipitation, fluorescence in situ hybridization assays, and rescue experiments were executed. We demonstrated that circDNAJC11 combined with TAF15 to promote BC progression via stabilizing MAPK6 mRNA and activating the MAPK signaling pathway.

**Conclusions:**

The circDNAJC11/TAF15/MAPK6 axis played a crucial role in the progression and development of BC, suggesting that circDNAJC11 might be a novel biomarker and therapeutical target for BC.

**Supplementary Information:**

The online version contains supplementary material available at 10.1186/s12967-023-04020-x.

## Background

Breast cancer (BC) has overtaken lung cancer as the most common cancer in the world, accounting for approximately 25% of new cancer cases and 15% of all cancer deaths in women [1, 2]. Despite tremendous advances that have been made in early diagnosis, chemoradiotherapy, and targeted therapy over the past decades, BC remains the leading cause of cancer death among women all over the world [3]. Therefore, there is an urgent need to find new biomarkers and effective potential targets for the diagnosis and treatment of BC.

In recent years, a new type of endogenous non-coding RNA, circular RNAs (circRNAs) have been discovered, characterized by a covalently closed loop structure without the 5’-cap and the 3’-poly A tail. CircRNAs have attracted much attention as important regulators, which are involved in the occurrence and development of a variety of human diseases, including cancer [4, 5]. CircRNAs function as miRNA sponges, RNA-binding protein scaffolds, regulators of splicing and transcription, as well as templates for protein translation [6]. Recently, some circRNAs have been reported to play crucial roles in BC tumorigenesis and progression. For instance, hsa_circ_001783 is significantly highly expressed and correlates with poor prognosis of BC patients. Hsa_circ_001783 promotes BC progression through sponging miR-200c-3p [7]. Additionally, Wang et al. have stated that hsa_circ_0005273 promotes BC tumorigenesis via sponging miR-200a-3p and inactivating the YAP1-hippo signaling pathway [8]. Another research has demonstrated that circNR3C2 is markedly downregulated in triple-negative breast cancer (TNBC) and negatively relates to distant metastasis. Upregulated circNR3C2 could increase the HRD1-mediated tumor-suppressive effects via sponging miR-513a-3p [9]. However, the biological functions of most circRNAs, especially their interactions with RNA binding protein (RBP) in BC remain largely elusive.

TATA-box-binding protein-associated factor 15 (TAF15) belongs to a conserved FUS-EWS-TAF15 (FET) family of RNA-binding proteins that play key a role in regulating gene expression, including polyadenylation, capping, RNA splicing, modification, localization, export, translation, and turnover [10, 11]. Accumulating evidence has indicated that TAF15 directly binds to and stabilizes lncRNAs and mRNAs to regulate the occurrence and development of various diseases. For instance, a recent study has demonstrated that LINC00504 recruits TAF15 to stabilize CPEB2 mRNA and enhance CPEB2 expression, thus decreasing radio-sensitivity of BC [12]. Meanwhile, Wang and his colleagues have found that lncRNA APOA1-AS increases proliferation and migration, and inhibits apoptosis of vascular smooth muscle cells via TAF15-mediated SMAD3 mRNA stabilization [13]. Besides, TAF15 has also been observed to stabilize LINC00665 in glioma cells via the STAU1-mediated mRNA degradation [14]. Moreover, lncRNA PITPNA-AS1 recruits TAF15 to stabilize HMGB3 mRNA to promote the proliferation and migration of lung squamous cell carcinoma cells [15]. However, the interaction between circRNAs and TAF15 in cancers is rarely reported.

Here, we first investigated the expression profile of circRNAs by a circRNA microarray and identified a novel BC-related circRNA, namely circDNAJC11, from DNAJC11 with a circBase ID of hsa_circ_0008389. Next, the clinical significance of circDNAJC11 expression, and its function and underlying mechanism in BC progression were explored. The results showed that circDNAJC11 was markedly up-regulated in BC cells and tissues and positively correlated with advanced tumor stage and poor prognosis. Further in vitro and in vivo functional and mechanistic experiments displayed that circDNAJC11 could remarkably increase BC cell proliferation, migration, invasion, and growth while inhibiting cell apoptosis by interacting with TAF15 to stabilize MAPK6 expression and activating the MAPK signaling pathway. Collectively, our data suggest that circDNAJC11 could serve as an oncogenic gene in BC progression and might be a promising marker and novel therapeutic target for BC.

## Methods

### Cell lines

Human BC cell lines (MCF-7, MDA-MB-231, BT-549, MDA-MB-453, and SK-BR-3) and normal breast epithelial cell lines (MCF-10A) were available from the American Type Culture Collection (ATCC, Manassas, VA, USA). For these cells, MCF-7, MDA-MB-231, and MDA-MB-453 cells were cultivated in DMEM (Gibco, Carlsbad, CA, USA) supplemented with 10% fetal bovine serum (FBS), BT-549, and SK-BR-3 cells were cultivated in the 10% FBS-contained RPMI-1640 medium (Gibco), and MCF-10A cells were cultured in MEBM Bullet Kit (Lonza, Basel, Switzerland). All these cells were subjected to cultivation in a humidified incubator at 37 °C with 5% CO_2_.

### BC tissue collection

Eighty pairs of BC tissues and paracarcinoma tissues were harvested from patients diagnosed as BC in the First Affiliated Hospital of Chongqing Medical University (Chongqing, China). None of these BC patients received preoperative chemotherapy or radiotherapy. Before RNA extraction, the tissue samples were retained in liquid nitrogen. The present investigation was conducted in accordance with the ethical standards and the Declaration of Helsinki and approved by the Ethics Committee of Chongqing Medical University (No. 2022-K228). Informed consent from the patients has been obtained.

### Microarray analysis

Total RNA of 4 pairs of non-TNBC and paracarcinoma tissues were extracted with TRIzol reagent (Takara, Dalian, China), followed by the measurement of the RNA quality with Nanodrop 1000 spectrophotometer (Thermo Fisher Scientific, Waltham, MA, USA). Subsequently, the linear RNA was digested with RNase R. The expression profiles of circRNAs were assessed by Arraystar Human circRNA Array V2. Data analysis was processed by Shanghai OE Biotech. Co., Ltd. (Shanghai, China).

### Quantitative real-time PCR (qRT- PCR)

The total RNAs were extracted from cells and tissues under the instructions of the Trizol reagent (Invitrogen, Carlsbad, CA, USA), which were identified by Nanodrop and agarose gel electrophoresis. The total RNAs were synthesized into cDNA with the PrimeScript RT kit (Takara). TB Green Premium Ex Taq (Takara) was utilized to amplify the cDNA on a Bio-Rad CFX96 system (Bio-Rad, CA, USA). The primers of genes in the study are listed in Additional file 3: Table S1.

### Nucleocytoplasmic separation, RNase-R, and Actinomycin D (Act D)

The nucleus and cytoplasmic RNAs in MCF-7 cells were isolated by using the PARIS™ Kit (Life Technologies, Austin, Texas, USA). The total RNAs from BC cells were digested for 40 min at 3 U/μg at 37 °C using RNase R (Epicenter Biotechnologies, Madison, WI, USA). The BC cells were reacted for 24 h with 4 μg/ mL of Act D (Cell Signaling Technology, Beverly, MA, USA). Then, the expression levels of the target genes were measured using qRT-PCR.

### RNA ISH

A digoxin-labeled probe (Digoxin-5’- TTCAGCTCTTCAGAAGAGGCCTTGGGAT TGGTTCGCTGCT-3’-Di-goxin) was synthesized for evaluating the circDNAJC11 expression in a tissue microarray (Outdo Biotech, Shanghai, China) containing 269 BC tissues and 134 paracarcinoma tissues. The tissue microarray was dewaxed, rehydrated, digested with proteinase K, and hybridized with the circDNAJC11 probe at 45 °C for 13 h. Afterward, the issues were combined with a biotin-conjugated anti-digoxin antibody for incubation overnight at 4℃, followed by 3,3-diaminobenzidine (DAB) staining. CircDNAJC11 expression was quantified by multiplying the positive staining intensity score (strong = 3, medium = 2, weak = 1, and negative = 0) by the percentage of positive-stained cells (> 76% = 4, 51–75% = 3, 26–50% = 2, 5–25% = 1, and < 5% = 0).

### Plasmids, siRNAs, and cell transfection

The full-length linear sequence of circDNAJC11 was amplified and subsequently cloned into the pLC5-ciR vector (Geneseed, Guangzhou, China) for the construction of the circDNAJC11 overexpression vector. MAPK6 overexpression plasmid was provided by Hanbio (Shanghai, China). The siRNAs targeting genes were synthesized by Geenseed (Guangzhou, China). Sequences of siRNAs in the study are listed in Additional file 4: Table S2. Cell transfection was implemented following the requirements of Lipofectamine TM 3000 (Invitrogen).

### Cell counting kit 8 (CCK-8), colony formation, and 5-Ethynyl-2’-deoxyuridine (EdU) assays

The absorbance value of BC cells at the wavelength of 450 nm was recorded using CCK-8 at the indicated time points to plot the growth curves. In terms of the colony formation assay, BC cells (1000 cells/well) were seeded into 6-well plates, which were then routinely cultured for two weeks before their fixed staining. Cells were stained using EdU reagent and 4',6-diamidino-2-phenylindole (DAPI) to capture the cell proliferation signals under a fluorescence microscope (Leica, Wetzlar, Germany).

### Flow cytometric analysis

BC cells were resuspended and fixed with 70% ethanol overnight at 4 °C for the measurement of the cell cycle arrest by flow cytometry (Becon Dickinson FACS Calibur, NY, USA). Additionally, BC cells were treated with Annexin V-FITC/PI staining and a Cell Apoptosis Kit for the evaluation of cell apoptosis by flow cytometry (Becon Dickinson FACS Calibur).

### Transwell assay

For the migration assay, 500 μl complete medium was supplemented to the lower chamber of the Transwell chamber, and then 5 × 10^5^ BC cells were resuspended in serum-free medium and subsequently added to the upper chamber of the Transwell chamber (the pore of the Transwell was 8.0 μm, Corning, Corning, NY, USA and Labselect, Chongqing, China). Upon a 24-h incubation in an incubator with 5% CO_2_, the cells were fixed by 75% ethanol and stained with crystal violet solution. For the invasion experiment, 50 μl of Matrigel diluted by serum-free medium was firstly supplemented into the upper chamber of the Transwell chamber, which was placed at 37 °C for 1 h. The rest steps were the same as the migration experiment.

### Western blotting

Total proteins from BC cells were extracted with radioimmunoprecipitation assay (RIPA) lysis buffer containing phenylmethyl sulfonylfluoride (PMSF), followed by SDS-PAGE, and transferred to polyvinylidene difluoride (PVDF) membranes (Millipore, Billerica, MA, USA). After that, the PVDF membranes were blocked with 5% skimmed milk and incubated with the corresponding primary antibody: Bax (1:1000, Abcam, Cambridge, UK), Bcl-2 (1:1000, Abcam), MAPK6 (1:1000, Abcam), p-MAPK6 (1:1000, Abcam), p38 (1:1000, Cell Signaling Technology, Danvers, MA, USA), p-p38 (1:1000, Cell Signaling Technology), ERK (1:1000, Cell Signaling Technology), p-ERK (1:1000, Cell Signaling Technology), and β-actin (1:5000, Cell Signaling Technology). Next, the membranes were incubated at 4℃ for 14 h, and subsequently, the membranes were incubated with the corresponding secondary antibody (1:6000, Cell Signaling Technology) for 1.5 h at room temperature. Lastly, the band signal was visualized using an enhanced chemiluminescence (ECL) detection system (Millipore).

### Immunohistochemistry (IHC)

The paraffin-embedded tissue sections were subjected to dewaxing, rehydration, as well as antigen repair. Next, the tissue sections were incubated at 4 °C overnight with Ki67 (1:100, Cell Signaling Technology), MAPK6 (1:50, Abcam) and Caspase-7-specific antibody (1:500, Abcam) followed by 1-h incubation at 37 °C with the biotin-labeled secondary antibody. Lastly, the tissues were stained with DAB and hematoxylin.

### Fluorescence in situ hybridization (FISH) and immunofluorescence (IF) assay

BC cells seeded on coverslips were subjected to incubation overnight at 4℃ with the corresponding specific antibody (TAF15, 1:200, Abcam) and with FITC-conjugated secondary antibody (1:200, Cell Signaling Technology) for 1 h at 37℃. Subsequently, the cells were cultivated for 13 h at 45℃ using a probe (5’CY3- AGAAGAGGCCTTGGGATTGGTTCGCTGC—3’CY3) (Geneseed) and then counterstained with DAPI.

### Biotinylated RNA pull-down assay and mass spectrometry

The RNA pull-down assay was implemented using a biotin-labeled circDNAJC11 probe (Genecreate, Wuhan, China) and a Pierce™ Magnetic RNA Protein Pull-Down Kit (Thermo Fisher Scientific). Finally, the interacting proteins were identified by mass spectrometry and western blot analysis.

### RNA immunoprecipitation (RIP) analysis

The RIP assays were conducted using the TAF15 (Abcam) and IgG (Abcam) control -specific antibodies, and the RNA Immunoprecipitation Kit (Geneseed). Expression levels of RNA and target protein in the samples were assessed by qRT-PCR and western blot, respectively.

### Animal experiments

Female BALB/c nude mice (4–6 weeks) were purchased from Chongqing Tengxin Biotechnology Co., Ltd. (Chongqing, China) and reared under the standard conditions of the Experimental Animal Center of Chongqing Medical University. MCF-7 cells (2 × 10^7^) at the logarithmic growth phase were resuspended in Matrigel (BD Biosciences, Bedford, MA, USA) and subcutaneously injected into the dorsal skin of mice. Tumor size was monitored for each mouse, which was calculated with the formula: length × width × width × 0.5. The tumor size was recorded after 28 days using a small animal imaging system (Berthold, Wildbad, Germany), and next, the mice were also euthanized. Finally, the excised tumors were weighed and measured. Meanwhile, a survival analysis was performed on mice (n = 10 mice/per group) with 70 days as the cutoff. All animal studies were identified by the Ethics Committee of the First Affiliated Hospital of Chongqing Medical University (No. 2022-K228).

### Statistical analysis

Statistical analyses were performed using GraphPad Prism 7.0 (San Diego, CA, USA) and SPSS 22.0 software (IBM, SPSS, Chicago, IL, USA). Student's t-test and one-way analysis of variance (ANOVA) were utilized to compare differences between two or three groups, respectively. Correlation between groups was processed using the chi-square test. The receiver operating characteristic (ROC) curve was adopted for evaluating the diagnostic value of circDNAJC11 in BC. The survival rate was plotted by Kaplan–Meier method and evaluated by a log-rank test. The factors related to survival were identified by a multivariate Cox proportional risk regression model. A probability value less than 0.05 was considered statistically significant.

## Results

### Identification and characterization of circDNAJC11 in BC cells

**Fig. 1:**
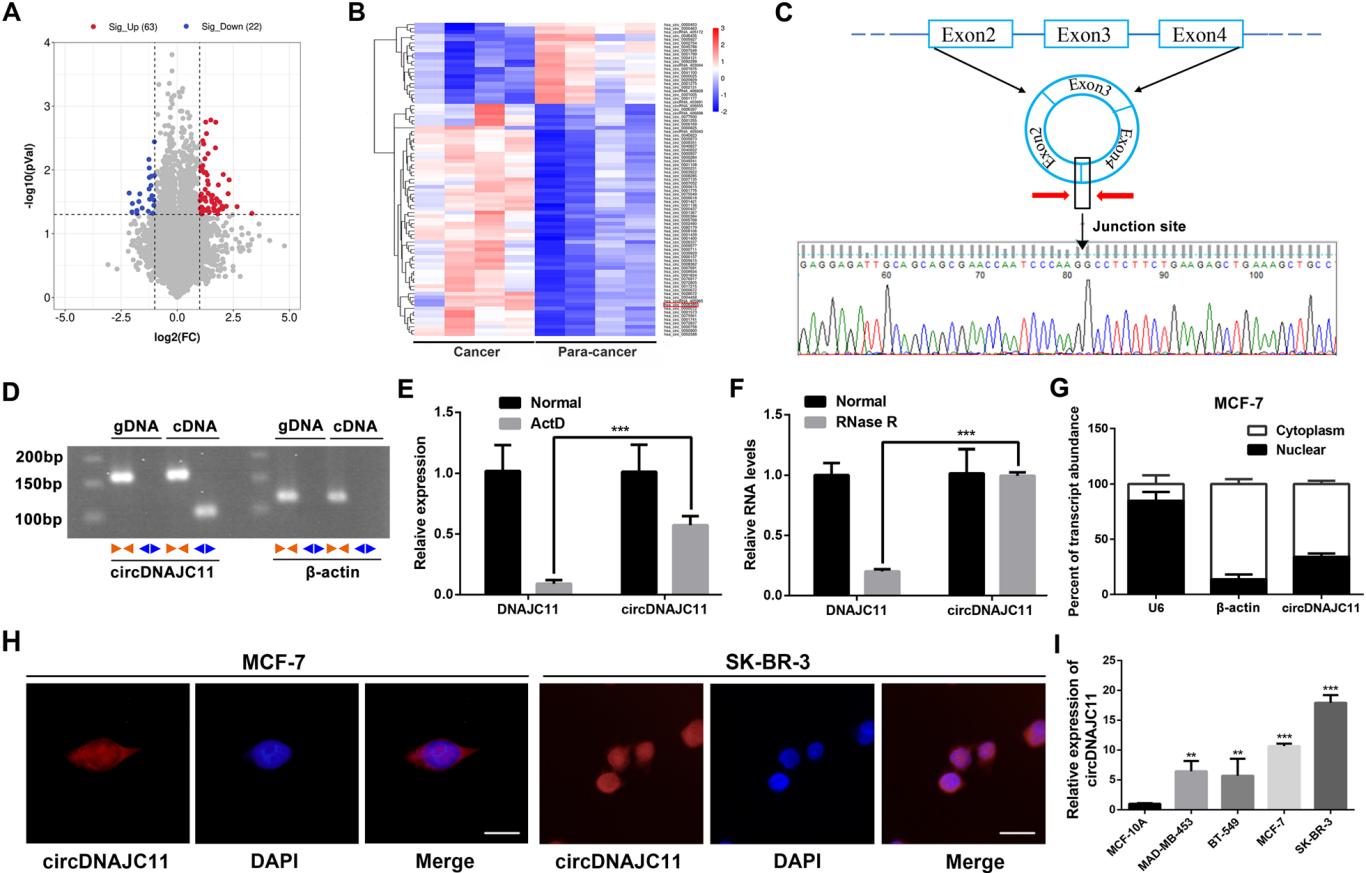
The identification of circDNAJC11. **A**, **B** Heatmap and scatter plot of the differentially expressed circRNAs in 4 paired BC tissues and adjacent non-tumor tissues using the RNA Microarray analysis. **C** Schematic representation of the DNAJC11 in the generation of the circDNAJC11. Sanger sequencing for the verification of the reverse splicing site of circDNAJC11. **D** CircDNAJC11 and β-actin were amplified from the cDNA or gDNA of MCF-7 cells using their divergent primers and convergent primers respectively. **E** The relative expression levels of circDNAJC11 and DNAJC11 mRNA were determined in MCF-7 cells with or without Act D treatment by qRT-PCR. **F** The relative expression levels of circDNAJC11 and DNAJC11 mRNA were evaluated in the Rnase R-treated RNA samples by qRT-PCR. **G** RNA nucleocytoplasmic separation combined with qRT-PCR was utilized to reveal the subcellular localization of circDNAJC11 in MCF-7 cells. **H** FISH assay was utilized to locate circDNAJC11 in subcellular fractions. Scale bar, 50 μm. **I** Relative expression of circDNAJC11 in BC cells and normal breast cells was determined by qRT-PCR. For **E**, **F**, and **I**, β-actin was utilized as a loading control. Data were presented as mean ± SD and representative of three independent experiments in **E**, **F**, **G**, and **I**. **E** and **F** were analyzed by Student’s t-test, and (I) was analyzed with ANOVA. **P < 0.01, ***P < 0.001

Differentially expressed circRNAs in BC tissues and their corresponding non-tumor tissues were analyzed by Microarray. With Fold change ≥ 2 and *P* < 0.05 as the screening criteria, 22 circRNAs were found to be up-regulated and 63 circRNAs were down-regulated in BC tissues (Fig. 1A, B). The circBank database and the UCSC database demonstrated circDNAJC11 as a circular RNA formed by the reverse splicing of the 2, 3 and 4 exon of the *DNAJC11* gene on chromosome 1 (Fig. 1C). Next, we designed specific divergent and convergent primers for the looping sites of circDNAJC11. The cyclization site of the PCR products was clearly defined by Sanger sequencing. The qRT-PCR and agarose gel electrophoresis indicated that the divergent primers of β-actin could not amplify bands in cDNA and gDNA, whereas the divergent primers of circDNAJC11 could amplify bands in cDNA (Fig. 1D). Subsequently, we validated its circRNA characteristics by RNase R digestion and Actinomycin D (Act D) assays. The combination of Act D with qRT-PCR demonstrated that the relative remaining expression of circDNAJC11 was significantly higher than that of DNAJC11 mRNA after Act D treatment (Fig. 1E). The results of the Rnase R digestion assay revealed that compared with RNA samples without Rnase R treatment, there exhibited reduced DNAJC11 mRNA levels in RNA samples treated with Rnase R, while the circDNAJC11 expression level did not change significantly (Fig. 1F). In addition, we analyzed the localization of circDNAJC11 in BC cells through RNA nucleocytoplasmic separation and FISH assays and found that circDNAJC11 was mainly distributed in the cytoplasm (Fig. 1G, H). As demonstrated in qRT-PCR, circDNAJC11 expression was significantly increased in BC cells relative to normal breast cells, and MCF-7 and SK-BR-3 cells exhibited the most significantly expressed circDNAJC11, which were selected for subsequent studies (Fig. 1I).

### CircDNAJC11 is up-regulated in BC and associated with clinicopathological parameters

**Fig. 2:**
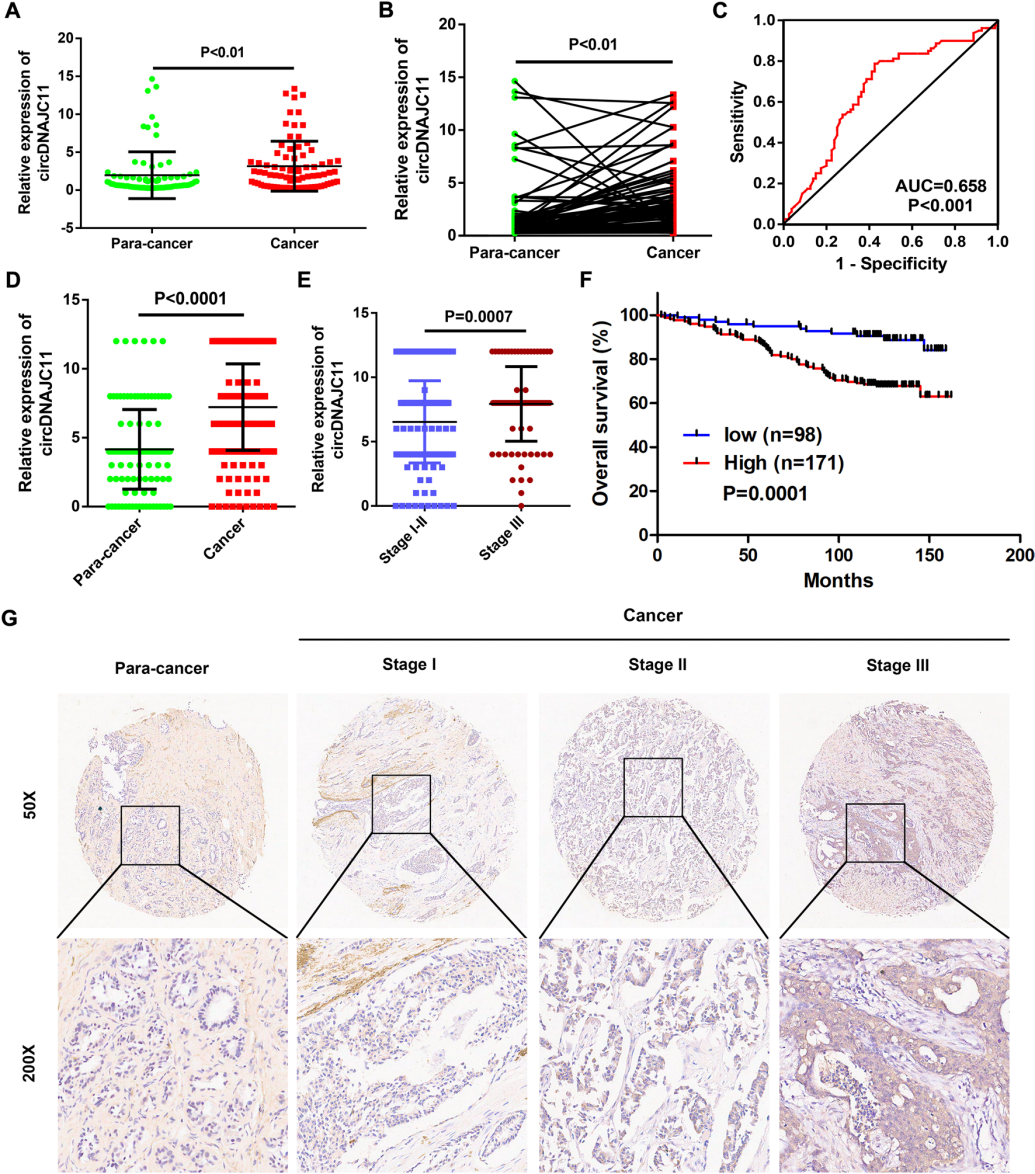
CircDNAJC11 is upregulated in BC tissues and is correlated with patient prognosis. **A** and **B** Relative expression of circDNAJC11 in BC and adjacent non-tumor tissues was determined by qRT-PCR. **C** The ROC curves for the assessment of the diagnostic value of circDNAJC11 in BC. **D** ISH scores of circDNAJC11 in BC tissues and para-cancer tissues on tissue microarray. **E** ISH scores of circDNAJC11 in BC tissues from patients at different stages on tissue microarray. **F** Kaplan–Meier curves for overall survival in different circDNAJC11 expression groups. Patients were divided into a high circDNAJC11 group and a low circDNAJC11 expression group based on the mean circDNAJC11 expression levels in BC tissues. **G** Representative images of ISH staining of circDNAJC11 on tissue microarray. For **A** and **B**, β-actin was utilized as a loading control. Data were presented as mean ± SD in **A**, **B**, **D**, and **E**. **A**, **B**, **D**, and **E** were analyzed with Student’s t-test, **C** was analyzed by ROC curve, and **F** was analyzed by log-rank test

Next, the determination of circDNAJC11 expression in 80 pairs of BC and adjacent non-tumor tissues by qRT-PCR suggested that circDNAJC11 was significantly overexpressed in BC tissues compared with adjacent non-tumor tissues (Fig. 2A and B). The ROC curve unveiled that circDNAJC11 had considerable diagnostic capacity in BC (Fig. 2C). Besides, the correlation analysis combined with the clinicopathological characteristics of patients revealed that circDNAJC11 expression in BC tissues was associated with T stage of BC patients (Table 1). Additionally, we also detected circDNAJC11 expression in the tissue microarray containing 269 BC tissues and 134 adjacent non-tumor tissues using ISH. A high expression level of circDNAJC11 was observed in BC tissues in comparison to the adjacent non-tumor tissues (Fig. 2D and G). CircDNAJC11 expression level in the tissues of patients at stage III was markedly higher than those patients at stage I-II (Fig. 2E). In the meantime, we found that circDNAJC11 expression in BC tissues was related to TNM stage following the clinicopathological characteristics and survival of patients, and the overall survival rate of patients with high expression of circDNAJC11 was remarkably lower than those with low expression (Table 2; Fig. 2F). Furthermore, multivariate cox regression models revealed that circDNAJC11 expression levels were independent risk factors affecting the prognosis of BC patients (Table 3). These results imply that circDNAJC11 may participate in the malignant progression of BC.

**Table 1:** The relationship between circDNAJC11 expression and the clinicopathological characteristics of 80 BC patients

Characteristics		circRNA		Chi-square	P value
		Low	High		
Age	< 50	14	24	0.013	0.908
	≥ 50	16	26		
Grade	I-II	18	21	2.432	0.119
	III	12	29		
T stage	T1	21	16	10.891	**0.001**
	T2-3	9	34		
N stage	N0	17	23	0.853	0.356
	N1-3	13	27		
TNM stage	I-II	12	30	3.008	0.083
	III	18	20

**Table 2:** Correlation between circDNAJC11 expression and clinicopathological features in 269 BC patients

Characteristics		circRNA		Chi-square	P value
		Low	High		
Age	< 50	42	62	1.144	0.285
	≥ 50	56	109		
Grade	I-II	82	145	0.060	0.807
	III	16	26		
T stage	T1	30	49	0.131	0.717
	T2-3	67	121		
N stage	N0	50	70	2.423	0.120
	N1-3	47	98		
TNM stage	I-II	78	99	12.400	**0.000**
	III	19	68

**Table 3:** Univariate and multivariate Cox regression analysis

Clinical variables	Univariate analysis		*P*	Multivariate analysis		*P*
	HR 95%CI			HR 95%CI		
Age (≥ 50 vs. < 50)	1.550	0.917–2.621	0.102			
Grade (I–II vs. III)	1.814	1.016–3.238	**0.044**	1.694	0.937–3.016	0.081
T stage (T1 vs. T2/3)	1.591	0.880–2.875	0.124			
N stage (N0 vs. N1-3)	1.754	1.049–2.993	**0.032**	1.268	0.608–2.645	0.527
TNM stage (I–II vs. III)	2.295	1.405–3.746	**0.001**	1.542	0.759–3.133	0.231
circRNA (low vs. high)	3.342	1.746–6.399	**0.000**	2.868	1.476–5.570	**0.002**

### CircDNAJC11 promotes the malignant phenotype of BC

**Fig. 3:**
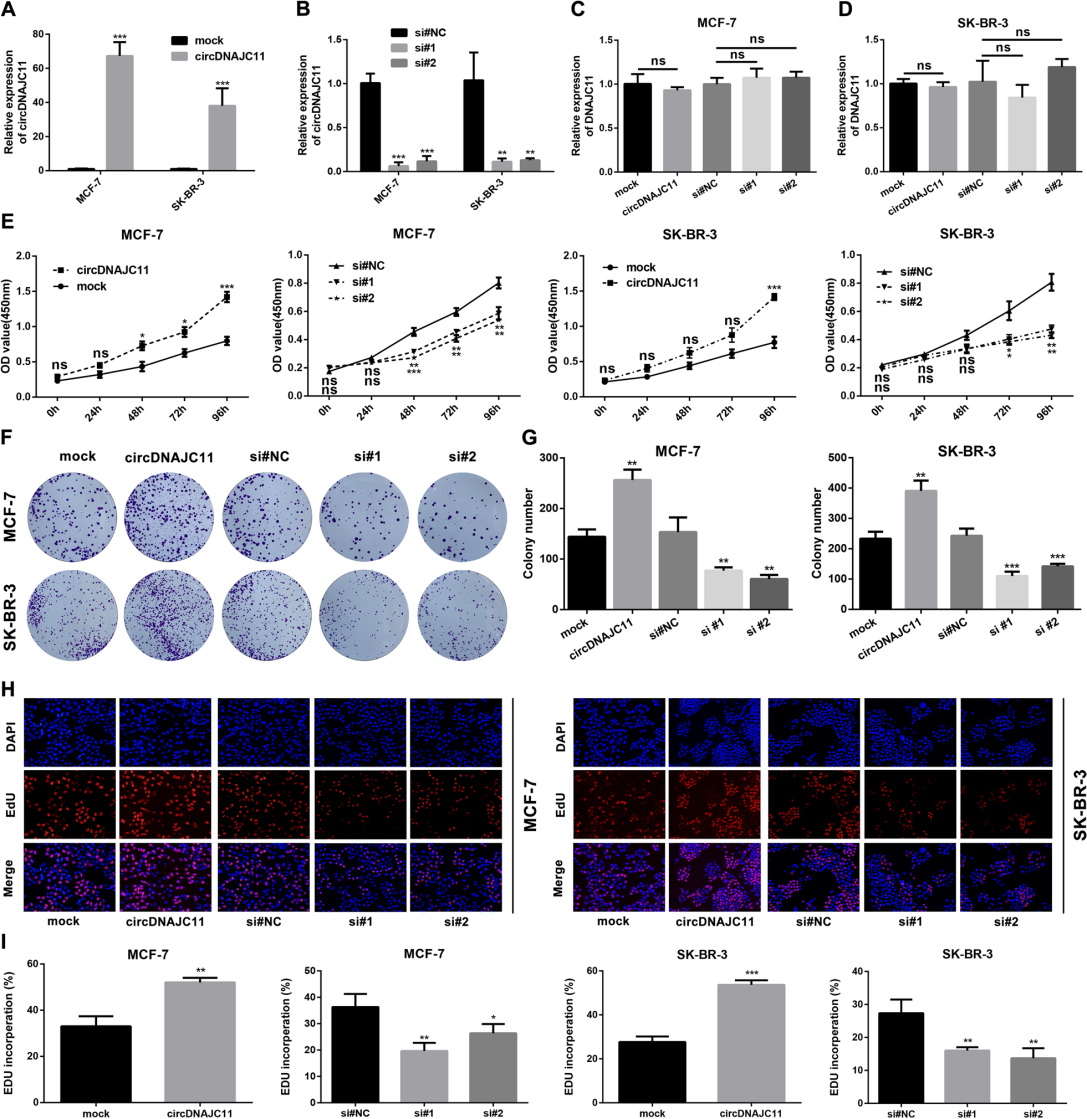
CircDNAJC11 promotes the proliferation of BC cells in vitro. **A**–**D** Relative expression of circDNAJC11 and DNAJC11 in BC cells transfected with indicated vectors and siRNAs was determined by qRT-PCR. **E** The proliferative activity of the cells was measured using CCK-8 assay after overexpression or knockdown of circDNAJC11 expression in MCF-7 and SK-BR-3 cells. **F** and **G** The effect of circDNAJC11 on the proliferative capacity of MCF-7 and SK-BR-3 cells was analyzed using a colony formation assay. The left panel **F** was the representative figure, and the right panel **G** showed the statistical analysis. **H** and **I** EdU experiment was implemented to evaluate the effect of overexpression or knockdown of circDNAJC11 on the malignant proliferation of BC cells (upper panel) and statistical analysis (lower panel). For **A**–**D**, β-actin was utilized a loading control. Data were presented as mean ± SD and representative of three independent experiments in **A**–**E**, **G**, and **I**. For the above experiments, the Student's t-test was used for comparison between two groups, and the ANOVA test was used for three groups. * P < 0.05, ** P < 0.01, ***P < 0.001, ns, no significance

**Fig. 4:**
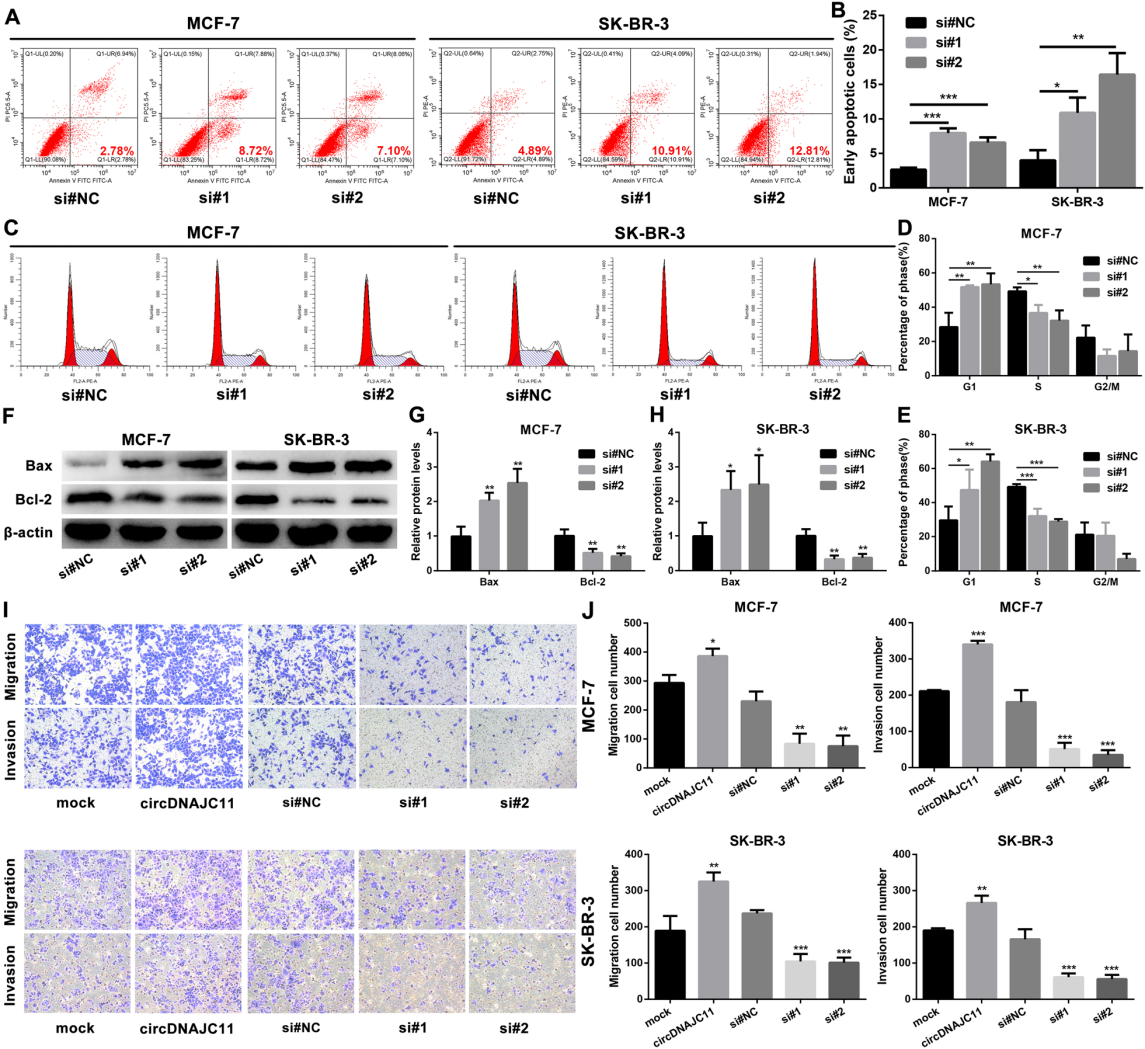
circDNAJC11 modulates BC cell apoptosis, cell cycle changes, and migratory and invasive capabilities. **A**–**E** BC cell apoptosis and cell cycle changes after circDNAJC11 knockdown were measured by flow cytometry. **F**–**H** After BC cells were transfected with corresponding siRNAs for 48 h, the expression levels of apoptosis-related proteins (Bax and Bcl-2) in BC cells were tested by western blot. **F** was the representative bands, and **G** and **H** were the statistical analysis. **I** and **J** Changes in migratory and invasive capabilities of MCF-7 and SK-BR-3 cells under different transfection conditions were analyzed by Transwell assay. **I** and **J** were the representative figures and the statistical analysis, respectively. Data were presented as mean ± SD and representative of three independent experiments in **B**, **D**, **E**, **G**, **H**, and **J**. For the above experiments, the Student's t-test was used for comparison between two groups, and the ANOVA test was used for three groups. * P < 0.05, ** P < 0.01, ***P < 0.001

To further reveal the biological functions of circDNAJC11 in BC, we initially constructed the circDNAJC11 overexpression vector and synthesized the siRNA against its reverse splice site to overexpress or knock down circDNAJC11 in cells. The qRT-PCR results disclosed that the circDNAJC11 overexpression vector effectively upregulated the circDNAJC11 expression in cells, and the two siRNAs could also significantly knock down the circDNAJC11 expression in cells, while neither the circDNAJC11 overexpression vector nor the siRNAs affected the DNAJC11 mRNA levels in cells (Fig. 3A–D). Subsequently, we performed CCK-8, colony formation, and EdU assays to probe into the impacts of circDNAJC11 on BC cell proliferation, and the findings unveiled that cell proliferative capacity was significantly enhanced in the circDNAJC11 overexpressing group while was diminished in the circDNAJC11 siRNA group in comparison to the control group (Fig. 3E–I). The findings of flow cytometry unveiled that compared with the control group, the cell apoptosis rate in the circDNAJC11 siRNA groups was significantly increased, and the cell cycle was blocked in the G0-G1 phase (Fig. 4A–E). Furthermore, we validated some of the apoptosis-related proteins using western blot and found a high Bax expression and a low Bcl-2 expression in cells of the circDNAJC11 siRNA groups compared to the control group (Fig. 4F–H). In the meantime, the Transwell migration and invasion assay demonstrated that the number of BC cells passing through the membrane increased significantly after overexpression of circDNAJC11, while after the knockdown of circDNAJC11, the number of BC cells passing through the membrane decreased significantly (Fig. 4I and J). These experimental results imply that circDNAJC11 promotes BC cell proliferative, migratory, and invasive capabilities, and arrests cells at G1 phase, while inhibiting apoptosis.

### CircDNAJC11 facilitates the growth of xenograft tumors

**Fig. 5:**
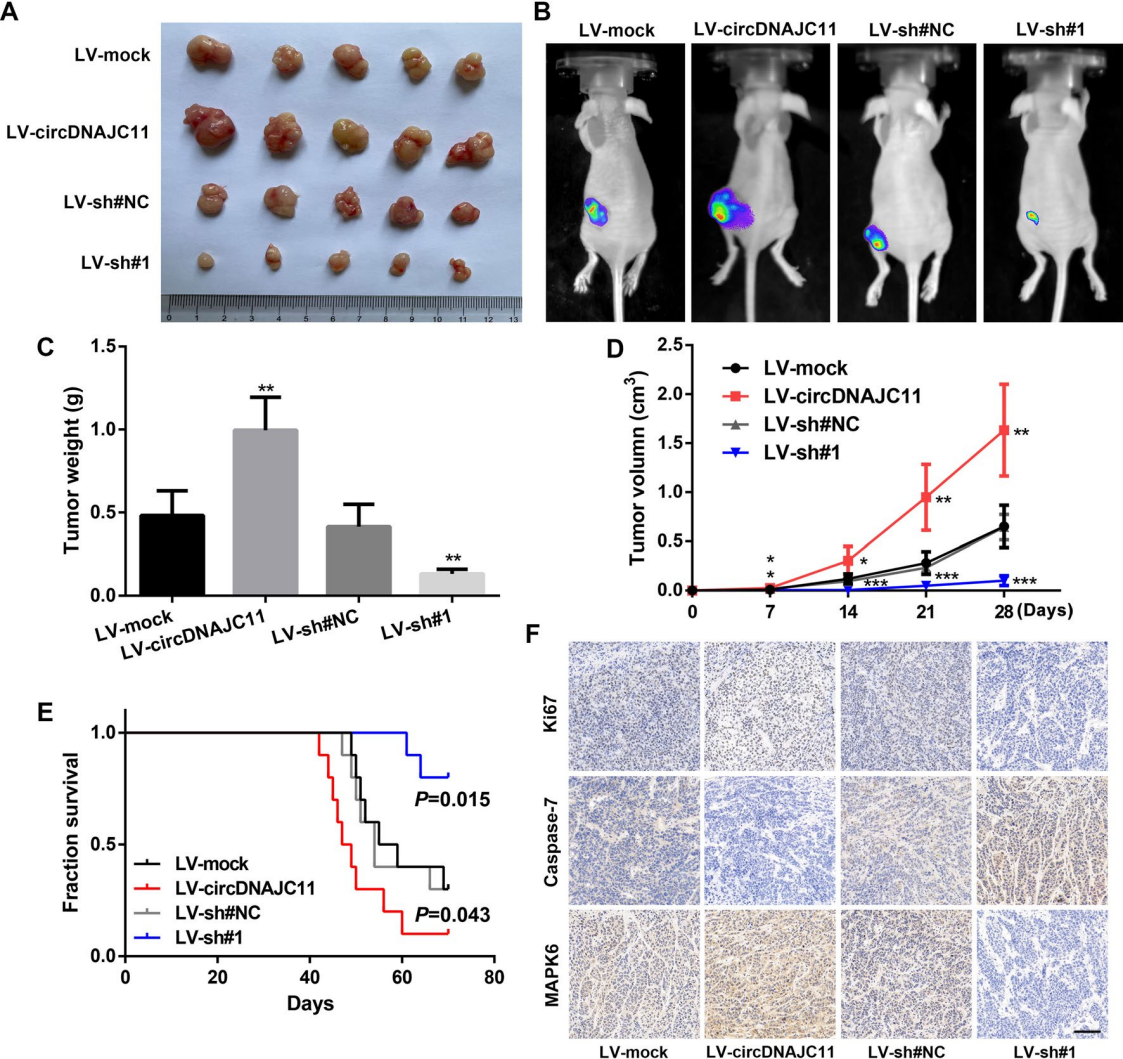
CircDNAJC11 promotes proliferation and regulates apoptosis of BC cells in vivo. **A** Representative figures of nude mice in each group (n = 5). **B** Representative bioluminescence images of the subcutaneous tumor model of nude mice in each group. **C** Comparison of the tumor weight in each group (n = 5). **D** Tumor xenograft growth curves for nude mice in each group were measured weekly (n = 5). **E** Kaplan–Meier survival curves for tumor-bearing mice inoculated with indicated stable MCF-7 cells (n = 10). **F** Immunohistochemistry for the examination of the expression levels of Ki67, MAPK6, and Caspase-7 in xenograft tumors. Scale bar, 100 μm. Data were presented as mean ± SD in **C** and **D**. **C** and **D** were analyzed with Student’s t-test, and **E** was analyzed with the log-rank test. * P < 0.05, ** P < 0.01, ***P < 0.001

Next, in vivo experiments were carried out to further verify the influences of circDNAJC11 on the biological functions of BC cells. First, circDNAJC11 overexpression and interference lentivirus were utilized to infect MCF-7 cells for the construction of MCF-7 cells that stably overexpress and knockdown circDNAJC11 (Additional file 1: Fig. S1A and B). The subcutaneous tumorigenesis experiment in nude mice indicated that compared with the cells infected with the corresponding control lentivirus, the tumor volume, weight, and growth rate increased significantly in cells infected with circDNAJC11 overexpression lentivirus, while the MCF-7 cells infected with circDNAJC11 knockdown lentivirus exhibited the opposite tendency, indicating that circDNAJC11 could enhance the tumorigenicity of MCF-7 cells in vivo (Fig. 5A–D). Meanwhile, the effect of circDNAJC11 on the survival of nude mice was also analyzed, and the overall survival rate of the nude mice was significantly lower in the circDNAJC11 overexpression group, and it was higher in the LV-sh#1 group in comparison to that in the control group (Fig. 5E). Next, we examined the expression levels of some key proteins in each group by immunohistochemistry. The findings demonstrated that circDNAJC11 overexpression effectively upregulated the expression levels of Ki67 and MAPK6, and down-regulated the expression of Caspase-7, while silencing circDNAJC11 played the opposite role (Fig. 5F). These functional experiments in vitro and in vivo suggest that circDNAJC11 promotes BC progression.

### CircDNAJC11 directly interacts with TAF15 and stabilizes MAPK6 and activates the MAPK signaling pathway

**Fig. 6:**
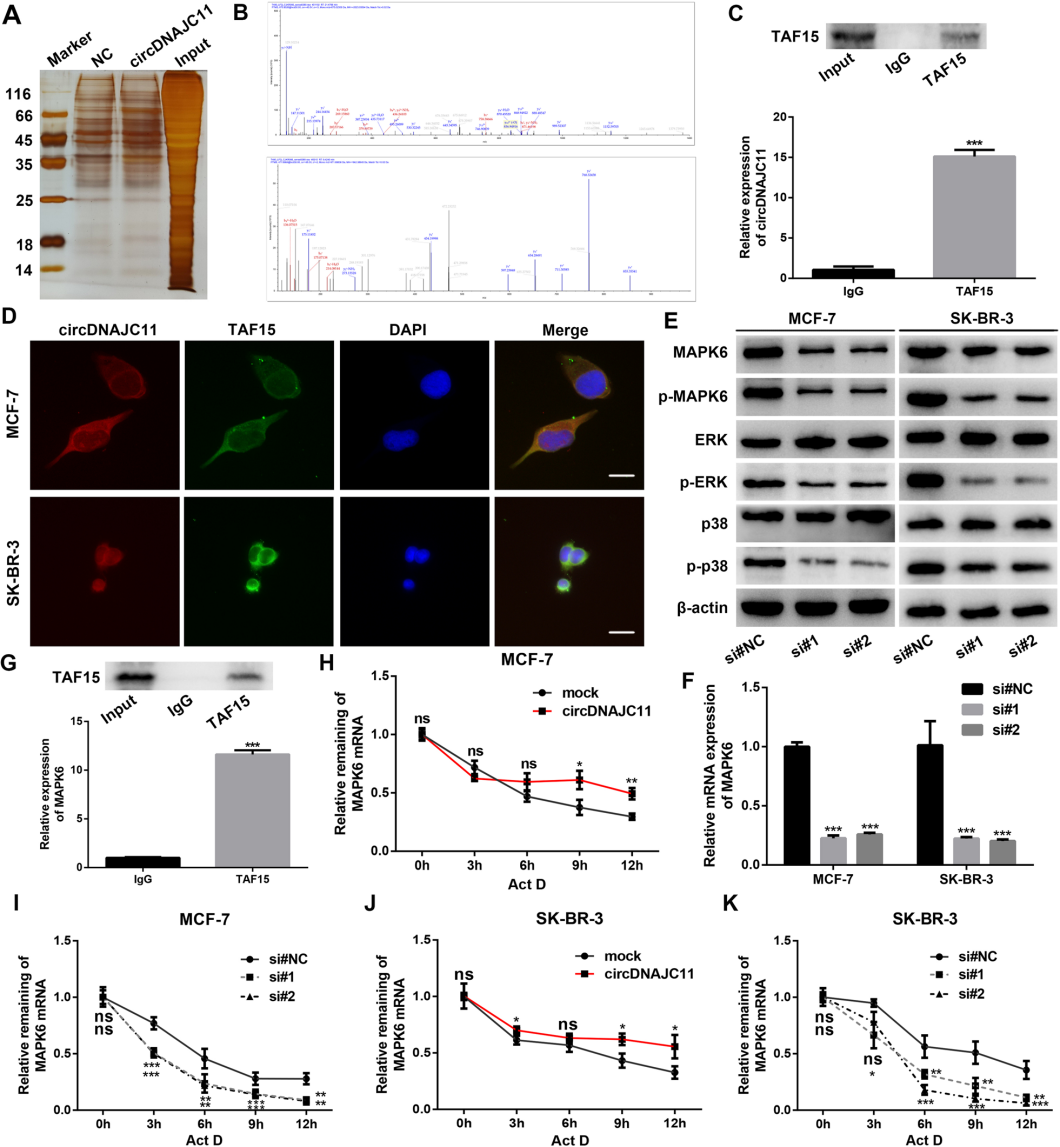
CircDNAJC11 modulates MAPK6 expression via TAF15. **A** Silver staining plot of proteins in MCF-7 cells was pulled down from the biotin-labeled circDNAJC11 probes and the control probes. **B** TAF15 peptide was determined by mass spectrometry analysis. **C** Binding of circDNAJC11 to TAF15 was determined by RIP assay using an anti-TAF15 antibody and an IgG control antibody. **D** FISH and IF double staining showing the localization of circDNAJC11 (red) and TAF15 (green) in MCF-7 and SK-BR-3 cells. Scale bar, 50 μm. **E** After BC cells were transfected with corresponding siRNAs for 48 h, the expression levels of the MAPK6 signaling pathway-associated proteins in BC cells of each group were evaluated by western blot. **F** The mRNA expression levels of MAPK6 in MCF-7 and SK-BR-3 cells transfected with si#NC or siRNAs for circDNAJC11 were assessed by qRT-PCR. **G** RIP experiments confirmed the binding of TAF15 protein and MAPK6 mRNA. **H**–**K** The effect of knockdown or overexpression of circDNAJC11 on MAPK6 mRNA stability in the presence of transcription inhibition using ActD (4 μg/mL) was determined by qRT-PCR. For **F**, β-actin was utilized as a loading control. Data were presented as mean ± SD and representative of three independent experiments in **C**, **F**, and **G**–**K**. **C**, **G**, **H**, and **J** were analyzed by Student’s-t test, and **F**, **I**, and **K** were analyzed by ANOVA. * P < 0.05, ** P < 0.01, ***P < 0.001, ns, no significance

Subsequently, to further elucidate the mechanism through which circDNAJC11 affected the progress of BC cells, we designed and synthesized a specific biotin-labeled probe for the reverse splicing site of circDNAJC11, and enriched the protein that may bind to circDNAJC11 by RNA pull-down in combination with LC–MS/MS experiments (Fig. 6A). It was found that TAF15 was significantly enriched by the circDNAJC11 probe, and two TAF15 peptides were identified (Fig. 6B). For further verifying the RNA pull-down results, we used the TAF15 antibody to carry out the RIP assay, which confirmed that the TAF15 antibody could effectively precipitate TAF15 protein and circDNAJC11, indicating the binding of circDNAJC11 and TAF15 (Fig. 6C). Next, FISH and IF double staining experiments were implemented to evaluate the distribution of circDNAJC11 and TAF15 in BC cells, revealing that the location of circDNAJC11 and TAF15 is consistent, which supports their combination (Fig. 6D). Recently, it has been reported that TAF15 affects MAPK6 expression by stabilizing MAPK6 mRNA in lung squamous cell carcinoma (LUSC) cells [16]. Therefore, we first tested the effect of circDNAJC11 on the mRNA and protein expression of MAPK6 in BC cells. The qRT-PCR and western blot results disclosed that circDNAJC11 knockdown decreased the mRNA and protein expression levels of MAPK6 when compared with the control group, and the opposite result was obtained after circDNAJC11 overexpression (Fig. 6E, F and Additional file 2: Fig. S2A–C). Meanwhile, we determined part of the MAPK signaling pathway-associated proteins and observed that circDNAJC11 was able to markedly modulate MAPK6, p-MAPK6, p-ERK, and p-p38 expression, while having no impact on the expression of non-phosphorylated ERK and p38 (Fig. 6E and Additional file 2: Fig. S2B, C). Furthermore, an interaction between TAF15 and MAPK6 mRNA was observed in BC cells by performing a RIP assay (Fig. 6G). Subsequently, BC cells were treated with Act D to examine the effect of circDNAJC11 on MAPK6 mRNA stability, which reflected that circDNAJC11 overexpression decreased the MAPK6 mRNA degradation rate, and circDNAJC11 knockdown increased it (Fig. 6H–K). Collectively, it is suggested that circDNAJC11 maintains MAPK6 mRNA stability through TAF15 to activate the MAPK6 signaling pathway.

### CircDNAJC11 promotes BC cell growth and metastasis via TAF15

**Fig. 7:**
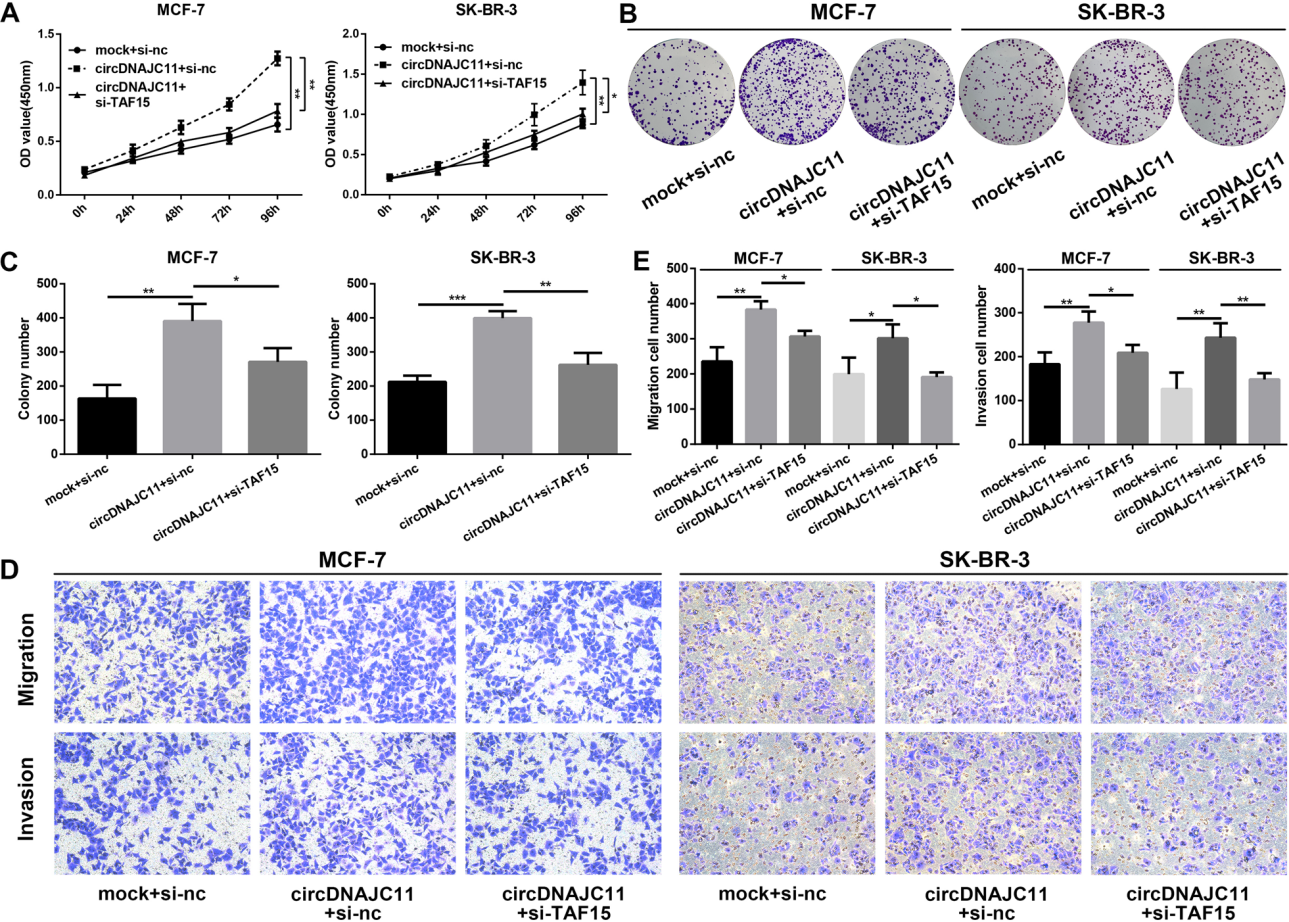
CircDNAJC11 induces BC proliferation, migration, and invasion through TAF15. (A-C) After BC cells were transfected with the corresponding overexpression plasmid or siRNAs for 48 h, CCK-8 (**A**) and colony formation (**B**, **C**) assay were performed to assess the cell proliferation of BC. **B** and **C** were representative pictures and statistical analyses of the colony formation assay, respectively. **D** and **E** The migration and invasion capabilities of the transfected BC cells in each group were determined using Transwell assay. **D** and **E** were representative pictures and statistical analyses of the Transwell assay, respectively. For **A**, **C**, and **E**, data were presented as means ± SD and analyzed using ANOVA, and experiments were repeated 3 times. * P < 0.05, ** P < 0.01, ***P < 0.001

Subsequently, to explore whether TAF15 mediated the promoted effect of circDNAJC11 on the progression of BC, we first up-regulated circDNAJC11 expression and knocked down TAF15 expression in BC cells to evaluate the ability of BC cells to proliferate and metastasize using CCK-8, colony formation, and Transwell assays. The findings of CCK-8 and colony formation assay disclosed that TAF15 down-regulation suppressed the enhanced cell proliferative capacity caused by circDNAJC11 overexpression (Fig. 7A–C). Meanwhile, the Transwell migration and invasion assay unveiled that circDNAJC11 overexpression enhanced the migration and invasion capabilities of BC cells, while this effect could be effectively suppressed by TAF15 knockdown (Fig. 7D and E). The results of the above experiments indicate that circDNAJC11 promotes the malignant phenotype of BC cells through TAF15, revealing that the circDNAJC11/TAF15/MAPK 6 signaling pathway plays a role in BC progression.

### CircDNAJC11 plays an oncogenic role in BC progression through MAPK6

**Fig. 8:**
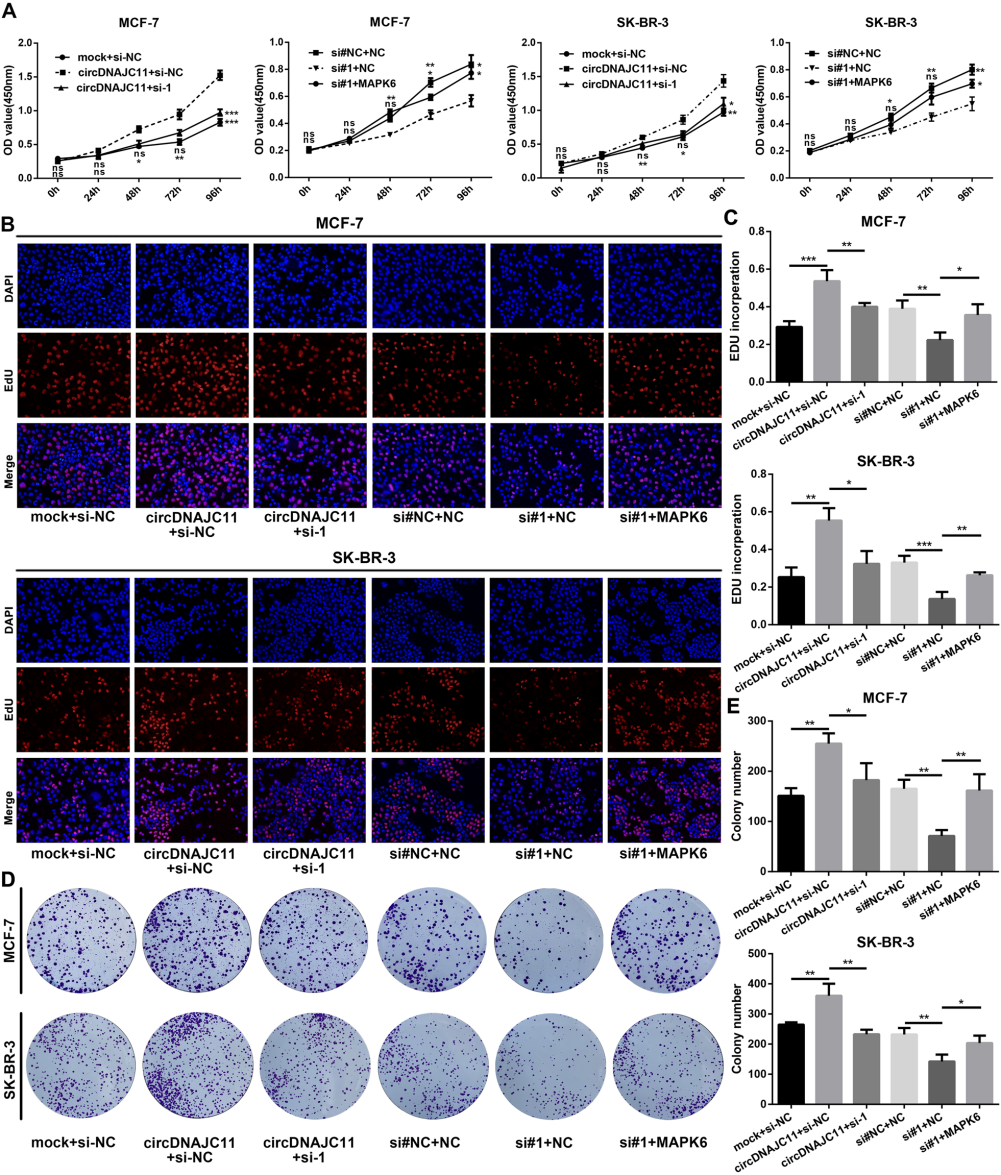
CircDNAJC11 affects the BC cell biological phenotype via MAPK6. **A** CCK-8 assay was performed to probe into the impacts of circDNAJC11/MAPK6 on BC cell proliferation. **B** and **C** A colony formation assay was implemented for determining the malignant proliferation level of BC cells transfected with the specified plasmid or siRNAs (**B**) and statistical analysis (**C**). **D** and **E** The malignant proliferation capacity of BC cells transfected with the indicated plasmid or siRNAs was determined by EdU assay. **D** and **E** were representative pictures and statistical analyses of the EdU assay, respectively. For **A**, **C**, and **E**, data were presented as means ± SD and analyzed using ANOVA, and experiments were repeated 3 times. * P < 0.05, ** P < 0.01, ***P < 0.001, ns, no significance

**Fig. 9:**
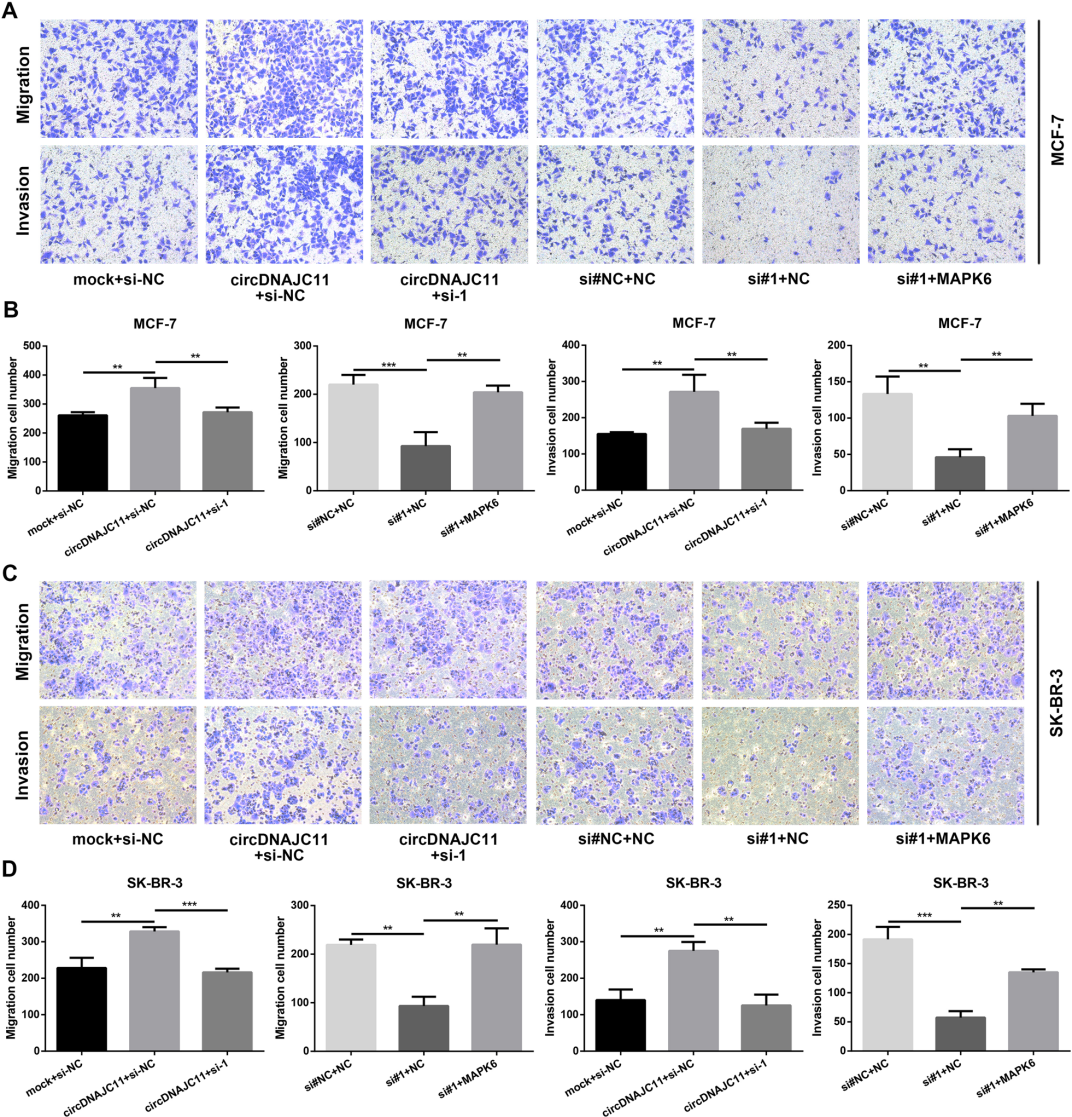
circDNAJC11 affects the BC cell migratory and invasive capabilities via MAPK6. **A** and **B** MCF-7 cell migratory and invasive capabilities under different transfection conditions were analyzed by Transwell assay. **A** and **B** were representative pictures and statistical analyses of the Transwell assay, respectively. **C** and **D** A Transwell assay was performed to evaluate the impact of the circDNAJC11/MAPK6 axis on SK-BR-3 cell migratory and invasive capabilities. **C** and **D** were representative pictures and statistical analyses of the Transwell assay, respectively. For **B** and **D**, data were presented as means ± SD and analyzed using ANOVA, and experiments were repeated 3 times. * P < 0.05, ** P < 0.01

To further confirm whether circDNAJC11 could influence the malignant phenotype of BC cells by enhancing MAPK6 expression, we conducted a series of rescue experiments. As demonstrated in the findings of CCK-8, colony formation, and EdU assays, MAPK6 knockdown reversed the enhancement of cell proliferation caused by circDNAJC11 overexpression, and MAPK6 overexpression reversed the reduction of cell proliferation caused by circDNAJC11 interference (Fig. 8A–E). In addition, Transwell assay results unveiled that the promoting effect of circDNAJC11 overexpression on BC cell migratory and invasive capacities was counteracted by the knockdown of MAPK6, while the inhibitory effect of knockdown of circDNAJC11 on BC cell migratory and invasive capacities was restored by upregulation of MAPK6 (Fig. 9A–D). The above data sufficiently suggest that circDNAJC11 accelerates BC progression by enhancing MAPK6 expression.

## Discussion

Accumulating evidence suggests that circRNAs play a crucial role in oncogenesis and progression through servicing as miRNA sponges, protein sponges, transcript regulators, and protein genes [17]. However, only a few circRNAs have currently been well studied and characterized, and their biological functions remain unclear. Importantly, our previous research illustrated that several circRNAs-interacting proteins could exert an oncogenic role in BC cells [18, 19], thus, we focused on the potential function of circDNAJC11 in BC. Here, with the help of the circRNA microarray analysis, we discovered that a novel circRNA, circDNAJC11, was highly expressed in BC tissue and cells. Next, clinical data showed that up-regulated circDNAJC11 was associated with the TNM stage and overall survival of BC patients. In functional assays, circDNAJC11 could promote BC cell proliferation, migration, and invasion in vitro, as well as tumor growth in vivo. Mechanistically, circDNAJC11 could bind to TAF15 to increase MAPK6 expression, thereby activating the MAPK signaling pathway and promoting BC progression.

As a new type of gene expression regulator, circRNAs appear in multiple biological processes. CircRNAs regulate gene expression by interacting with diverse cellular modulators, such as microRNAs and RNA-binding proteins (RBPs), to regulate the downstream gene expression [20]. Dysregulation of RBPs has been implicated in tumorigenesis and progression of cancers [21]. Although circRNAs exert their functions in a variety of ways, the vast majority of circRNAs are commonly studied as miRNAs sponge. In recent years, there have been increasing reports focusing on the interactions of circRNAs and proteins. For example, circRNA CDR1as might be directly combined with the p53 DBD domain structure, thus disintegrating the p53/MDM2 complex and inhibiting gliomagenesis [22]. CircACTN4 has been demonstrated to interact with YBX1 to activate FZD7 transcription, thereby promoting intrahepatic cholangiocarcinoma progression [23]. Meanwhile, circPOLR2A interacts with UBE3C and PEBP1 proteins and promotes the UBE3C-mediated PEBP1 ubiquitination, thereby activating the ERK signaling pathway and facilitating clear cell renal cell carcinoma progression [24]. According to RNA pull-down, mass spectrum, and RNA immunoprecipitation assays, we demonstrated that circDNAJC11 could bind to TAF15 and stabilize the expression of some mRNAs. Our experimental results support these findings. Collectively, these data indicated that circRNAs could bind to proteins for the modulation of the genesis and development of tumors.

TAF15, a member of the FET family consisting of TATA binding protein (TBP) and a set of evolutionarily conserved proteins involved in basic transcription, coordinates transcription initiation of RNA polymerase II , binds to the core promoter to correctly locate the polymerase, acts as a scaffold to assemble the remaining transcription complex, and acts as a pathway for regulating signaling [25, 26]. Except for functioning as a transcription factor, TAF15 also stabilizes mRNAs by binding to G-rich sequences in 3’-UTRs [14]. TAF15 plays an important role in regulating mRNA transcription, RNA splicing, and transportation in cancer and other diseases [27, 28]. Pan et al. have reported that TRPM2-AS promotes the proliferation of colorectal cancer cells by increasing the TAF15-mediated TRPM2 mRNA stability [29]. Moreover, research has demonstrated that circVMA21 binds with TAF15 to stabilize SOCS3 mRNA to alleviate septic lung injury by regulating the NF-κB activation [30]. Ren et al. confirmed that LncRNA PITPNA-AS1 recruited TAF15 to HMGB3 3’UTR, thus stabilizing HMGB3 mRNA. Recently, LINC00649 has been revealed to interact with TAF15 to facilitate the progression of lung squamous cell carcinoma by enhancing MAPK6 expression and activating the MAPK signaling pathway [16]. Consistent with the previous reports, our observations suggest that circDNAJC11 directly binds to TAF15 in the cytoplasm of BC. We also demonstrated that TAF15 was the key protein for circDNAJC11 to play a role in promoting the malignant phenotype of BC.

MAPK6, also known as ERK3, is an atypical MAPK. The MAPK6 pathway has been reported to be involved in the inflammatory response, cell growth, and differentiation [31]. Specifically, the MAPK signaling pathway has also been shown to influence the biological behaviors of cancers [32]. For instance, Lv et al. have demonstrated that MAPK6 is highly expressed in BC and is related to poor prognosis of BC patients [33]. Wu et al. have reported that MAPK6 expression is upregulated in non-small-cell-lung cancer cells [34]. Furthermore, another article has elucidated that the knockdown of MAPK6 suppresses cervical cancer cell proliferation, migration, and invasion [35]. In the present study, we found that circDNAJC11 could directly bind to TAF15 to effectively regulate the protein expression of MAPK6 to influence the MAPK signaling pathway. We assume that circDNAJC11 recruit TAF15 to MAPK6 3’UTR, thus stabilizing MAPK6 mRNA. Notably, circDNAJC11 is required to influence BC progression via MAPK6. This may be due to the fact that circDNAJC11 can bind and play a role in promoter recognition after interacting with TAF15 or modify general transcription factors (GTFs) to promote complex assembly and transcription initiation, thereby facilitating BC progression. In studies on other types of cancer, such as in colorectal cancer and bladder cancer cell lines, BRD4-bound circRNA regulates MYC expression [36, 37], and BRD4 bound the super-enhancer regulates expression levels of lncRNA PVT1 and MYC [38]. It has also been shown that interference with circDNA enhancer by CRISPR technology can effectively block the transcriptional activation pattern of this unique enhancer-gene [39]. Given the diverse functionality of circRNA [40], the details of the process that affects transcription upon binding of circDNAJC11 to TAF15 in this study deserve further investigation.

The circRNA-protein interactions to promote gene transcription beyond proto-oncology provide a new potential target for the treatment of cancer. Unlike genomic transcription, where genomic DNA enhancers mostly interact with each other as proximate regions, circRNA clusters are protein-interacting to form clusters that can act distally to promote gene expression and therefore have a greater range of influence [41]. In this study, we demonstrated that circDNAJC11 directly binds to TAF15 in the cytoplasm of BC and increases MAPK6 expression. More importantly, we validated the role of TAF15 in the TET family of RNA-binding proteins in circDNA and tumor development, which may also be associated with cancer and drug resistance.

The circRNA carries oncogenes and drug resistance genes, which can effectively increase the amplification efficiency and expression level of genes. Moreover, circRNA is randomly assigned during mitosis, which would result in the cancer driver genes it carries being mobile among tumor cells. The amount of circRNA changes with environmental conditions, for example, circ-EGFR in gliomas, especially oncogenes and drug resistance genes, and the amount of circRNA changes dynamically with different periods of division [42, 43]. Studies of circRNA in tumor cells have shown that circRNA is very common in cancer and has an important role in both tumor heterogeneity, adaptation and evolution [44]. Therefore, it is of great significance for other studies that interact with circRNA and generate interactions on tumorigenesis development, such as the interaction of circDNAJC11 with TAF15 in this study.

## Conclusion

In conclusion, this research highlights that circDNAJC11 is upregulated in BC and is associated with the prognosis of BC. Meanwhile, circDNAJC11 directly interacts with TAF15 to enhance the facilitation of TAF15 on the MAPK6 mRNA stability, thereby activating the MAPK signaling pathway and ultimately promoting BC progression. The findings may contribute to our understanding of the biological mechanisms by which circDNAJC11 promotes BC development, and highlight that circDNAJC11 may be a novel biomarker and therapeutic target for BC.

## Supplementary Information

**Additional file 1: Figure S1.** Effects of circDNAJC11 overexpression and knockdown lentivirus on circDNAJC11 expression. (A and B) The expression levels of circDNAJC11 in stably-transfected cell lines were determined using qRT-PCR. For (A) and (B), β-actin was utilized as a loading control. Data were presented as means ± SD and analyzed using Student’s t-test, and experiments were repeated 3 times. **P < 0.01, ***P < 0.001.

**Additional file 2: Figure S2.** CircDNAJC11 affects the MAPK6 signaling pathway. (A) The influence of circDNAJC11 overexpression on MAPK6 was determined by qRT-PCR. (B-C) The impact of circDNAJC11 knockdown on the MAPK6 signaling pathway-related proteins was assessed by western blot. For (A), β-actin was utilized as a loading control. Data were presented as mean ± SD and representative of three independent experiments in (A-C). (A) was analyzed by Student’s t-test, and (B) and (C) were analyzed by ANOVA. * P < 0.05, ** P < 0.01, ***P < 0.001, ns, no significance.

**Additional file 3: Table S1.** Sequences of primers used in this study.

**Additional file 4: Table S2.** Sequences of siRNAs used in this study.

## Data Availability

Not applicable.

## Abbreviations

circRNA: Circular RNA
BC: Breast cancer
TNBC: Triple-negative breast cancer
RBP: RNA binding protein
TAF15: TATA-box-binding protein-associated factor 15
FET: FUS-EWS-TAF15
DAB: Diaminobenzidine
DAPI: 4',6-Diamidino-2-phenylindole
PMSF: Phenylmethyl sulfonylfluoride
PVDF: Polyvinylidene difluoride
IHC: Immunohistochemistry
FISH: Fluorescence in situ hybridization
IF: Immunofluorescence
RIP: RNA immunoprecipitation
ANOVA: Analysis of variance
ROC: Receiver operating characteristic
Act D: Actinomycin D
RBPs: RNA-binding proteins
GTFs: General transcription factors
