# Supplementary figures and images for: CircDNAJC11 interacts with TAF15 to promote breast cancer progression via enhancing MAPK6 expression and activating the MAPK signaling pathway
Source: J Transl Med. 2023 Mar 9;21:186. doi: 10.1186/s12967-023-04020-x (PMC9999642; doi:10.1186/s12967-023-04020-x)

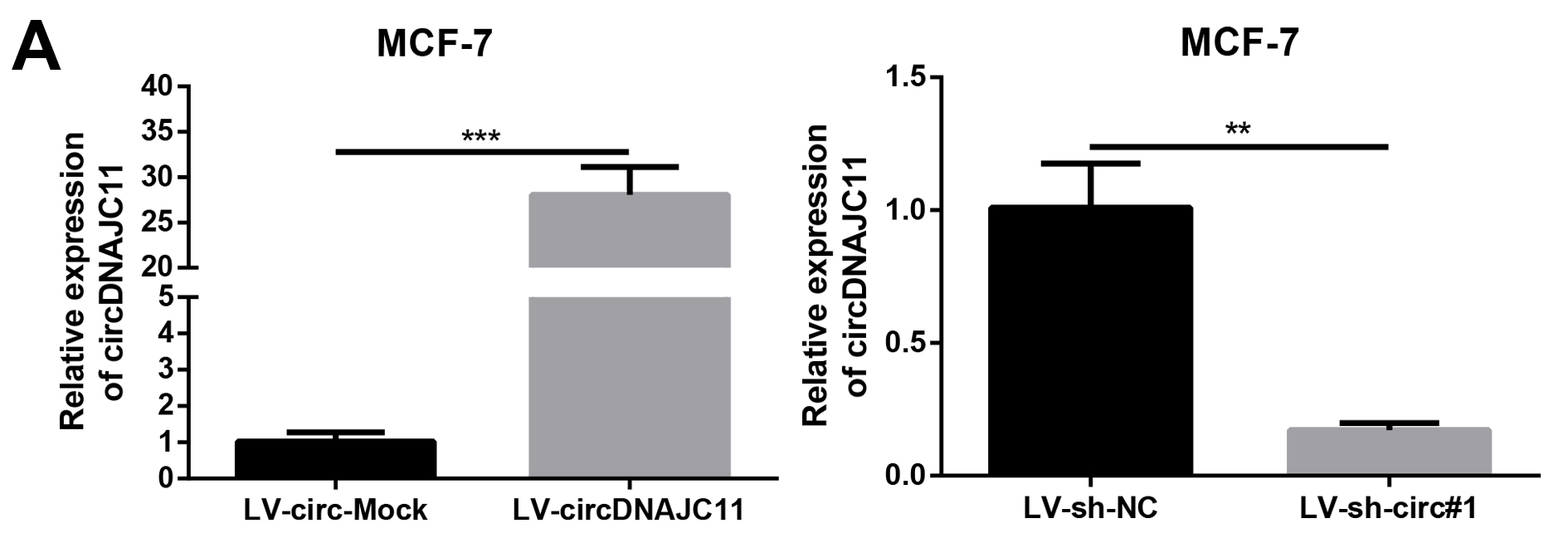

Supplement: Supplementary file 1 — Additional file 1: Figure S1. Effects of circDNAJC11 overexpression and knockdown lentivirus on circDNAJC11 expression. (A and B) The expression levels of circDNAJC11 in stably-transfected cell lines were determined using qRT-PCR. For (A) and (B), β-actin was utilized as a loading control. Data were presented as means ± SD and analyzed using Student’s t-test, and experiments were repeated 3 times. **P < 0.01, ***P < 0.001. [file 12967_2023_4020_MOESM1_ESM.jpg]

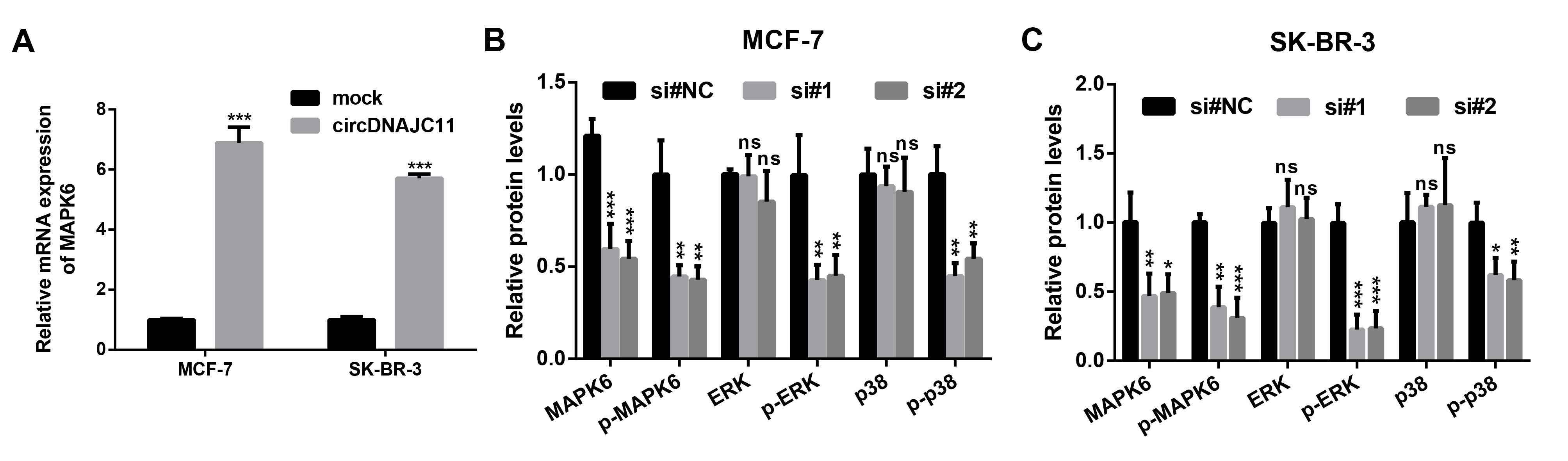

Supplement: Supplementary file 2 — Additional file 2: Figure S2. CircDNAJC11 affects the MAPK6 signaling pathway. (A) The influence of circDNAJC11 overexpression on MAPK6 was determined by qRT-PCR. (B-C) The impact of circDNAJC11 knockdown on the MAPK6 signaling pathway-related proteins was assessed by western blot. For (A), β-actin was utilized as a loading control. Data were presented as mean ± SD and representative of three independent experiments in (A-C). (A) was analyzed by Student’s t-test, and (B) and (C) were analyzed by ANOVA. * P < 0.05, ** P < 0.01, ***P < 0.001, ns, no significance. [file 12967_2023_4020_MOESM2_ESM.jpg]
