# Supplementary material for: CircDNAJC11 interacts with TAF15 to promote breast cancer progression via enhancing MAPK6 expression and activating the MAPK signaling pathway
Source: J Transl Med. 2023 Mar 9;21:186. doi: 10.1186/s12967-023-04020-x (PMC9999642; doi:10.1186/s12967-023-04020-x)
Supplement: Supplementary file 3 — Additional file 3: Table S1. Sequences of primers used in this study. [file 12967_2023_4020_MOESM3_ESM.docx]

**Table S1. Sequences of primers used in this study.**

| Gene | Primer sequences |
| --- | --- |
| circDNAJC11 (Divergent) | F: 5’-GAACCAATCCCAAGGCCTC-3’ |
|  | R: 5’-GACTTGAGCTCTGGGTCTCT-3’ |
| circDNAJC11 (Convergent) | F: 5’-ACAGAAGGCACTCACCACAA-3’ |
|  | R: 5’-CGGTAGGCACTTTCAGCTCT-3’ |
| β-actin (Convergent) | F: 5’-CATGTACGTTGCTATCCAGGC-3’ |
|  | R: 5’-CTCCTTAATGTCACGCACGAT-3’ |
| β-actin (Divergent) | F: 5’-AAATCGTGCGTGACATTAAGGAGA-3’ |
|  | R: 5’-CATACCCCTCGTAGATGGGCA-3’ |
| U6 | F: 5’-CTCGCTTCGGCAGCACA-3’ |
|  | R: 5’-AACGCTTCACGAATTTGCGT-3’ |
|  | RT: 5’-CTCGCTTCGGCAGCACAPCR-3’ |
| MAPK6 | F: 5’- AGCTTGGGGAGAGAGGACAT-3’ |
|  | R: 5’- GTGGGATGCCTATGGACTCG-3’ |
