# Supplementary material for: CircDNAJC11 interacts with TAF15 to promote breast cancer progression via enhancing MAPK6 expression and activating the MAPK signaling pathway
Source: J Transl Med. 2023 Mar 9;21:186. doi: 10.1186/s12967-023-04020-x (PMC9999642; doi:10.1186/s12967-023-04020-x)
Supplement: Supplementary file 4 — Additional file 4: Table S2. Sequences of siRNAs used in this study. [file 12967_2023_4020_MOESM4_ESM.docx]

**Table S2. Sequences of siRNAs used in this study.**

| Definition | sequences |
| --- | --- |
| si#NC | 5’-TTCTCCGAACGTGTCACGTAA-3’ |
| si#1(circDNAJC11) | 5’-ATCCCAAGGCCTCTTCTGA-3’ |
| si#2(circDNAJC11) | 5’-TCCCAAGGCCTCTTCTGAA-3’ |
| si-1(MAPK6) | 5’-GAGCTCTGTCCGATGTCACTGATGA-3’ |
| si-TAF15 | 5’-AACAGATGCTGATTCAGAATCTGAT-3’ |
